# Revealing the acute asthma ignorome: characterization and validation of uninvestigated gene networks

**DOI:** 10.1038/srep24647

**Published:** 2016-04-21

**Authors:** Michela Riba, Jose Manuel Garcia Manteiga, Berislav Bošnjak, Davide Cittaro, Pavol Mikolka, Connie Le, Michelle M. Epstein, Elia Stupka

**Affiliations:** 1 Center for Translational Genomics and Bioinformatics, San Raffaele Scientific Institute, Milan, Italy; 2 Experimental Allergy Lab, Department of Dermatology, DIAID, Medical University of Vienna, Austria

## Abstract

Systems biology provides opportunities to fully understand the genes and pathways in disease pathogenesis. We used literature knowledge and unbiased multiple data meta-analysis paradigms to analyze microarray datasets across different mouse strains and acute allergic asthma models. Our combined gene-driven and pathway-driven strategies generated a stringent signature list totaling 933 genes with 41% (440) asthma-annotated genes and 59% (493) ignorome genes, not previously associated with asthma. Within the list, we identified inflammation, circadian rhythm, lung-specific insult response, stem cell proliferation domains, hubs, peripheral genes, and super-connectors that link the biological domains (*Il6, Il1ß, Cd4, Cd44, Stat1, Traf6, Rela, Cadm1, Nr3c1, Prkcd, Vwf, Erbb2*). In conclusion, this novel bioinformatics approach will be a powerful strategy for clinical and across species data analysis that allows for the validation of experimental models and might lead to the discovery of novel mechanistic insights in asthma.

Allergen exposure causes a complex interaction of cellular and molecular networks leading to allergic asthma in susceptible individuals. Experimental mouse models of allergic asthma are widely used to understand disease pathogenesis and elucidate mechanisms underlying the initiation of allergic asthma^1^. For example, gene profiling of lung tissue from experimental mice during the initiation of allergic asthma in experiments with different protocols^2^,^3^,^4^,^5^,^6^,^7^ validated well-known genes and identified new genes with roles in disease pathogenesis such as *C5*^3^, *Arg1*^8^, *Adam8*^9^, and *Pon1*^7^, and dissected pathways activated by *Il13*^10^ and *Stat6*^11^. Despite these datasets revealing large numbers of genes expressed in lungs during the onset of allergic asthma, there is a skewed distribution of research and literature coverage on a small number of genes, as has been noted in other fields^12^,^13^,^14^.

Previous meta-analysis of asthma datasets generated either very small^15^ or very large gene lists^16^ using approaches based on the analysis of gene lists. There is a need for new approaches that generate testable gene lists. To fully understand the genes and pathways in the lung that are involved in the initiation of allergic asthma, we reviewed all 23 published gene expression experiments performed on lungs from mice with allergic asthma to select those of sufficient robustness to be utilized for a multi-dataset bioinformatics analysis. We focused on 6 studies that share a common microarray platform, have a robust disease phenotype and were derived from experiments using different mouse strains and experimental protocols^2^,^3^,^4^,^5^,^6^,^7^. We used a novel bioinformatics strategy combining gene-driven and pathway-driven approaches (see methods) to generate a stringent asthma signature list. This list included a large set of protein-coding genes that had no published function or literature connecting them to allergic asthma either at the level of the whole organism or for individual organs, tissues or cells, genes for which the term “ignorome” was recently coined^17^. Here, we present a gene signature list from mice consisting of 933 genes with 440 asthma-annotated and 493 “acute allergic asthma ignorome” genes. This novel bioinformatics approach may also be powerful when used on large datasets from clinical studies, allowing for comparisons between mouse and human datasets thus, leading to the discovery of disease-specific genes and pathways.

## Results

### Acute allergic asthma gene signature list

**Figure 1:**
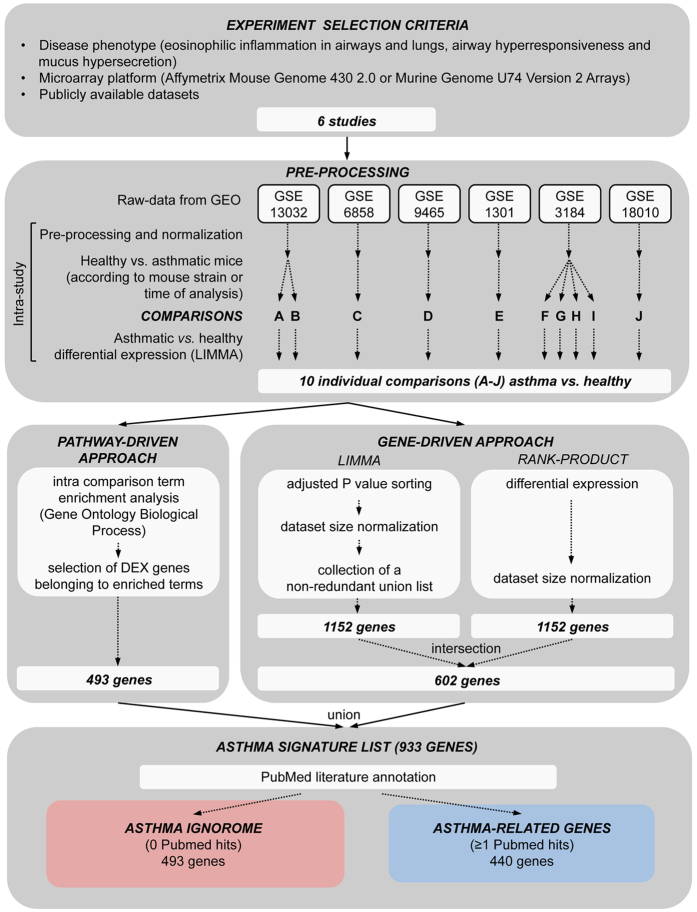
Schematic overview of analysis pipeline. Using experiment selection criteria, we selected six studies that were further subdivided into 10 comparisons of control and asthmatic mice according to mouse strain and time of analysis. After initial analysis with liner models to obtain differential gene expression, data from each comparison were re-analyzed using pathway- and gene-driven approaches (for details please refer to supplementary methods). Lists of differentially regulated genes generated in the 2 approaches were merged into a final asthma signature list of 933 genes, which was used for literature-coverage searches in PubMed.

To select the datasets for analysis, we analysed 23 microarray studies of lung RNA from mice at the initiation of allergic asthma [Ref. 18 and GEO database (http://www.ncbi.nlm.nih.gov/geo/)]. Our selection criteria included the phenotype elicited and data robustness (see methods). Six microarray datasets [Refs 2,10,19,20 and GSE1301 and GSE3184] from 5 different mouse strains, 2 allergens, 6 experimental protocols were subdivided into 10 comparisons of control *vs.* asthmatic mice (A–J) (Table 1 and Fig. 1).

To create an acute asthma signature list from these 6 microarray datasets, we used a multi-dataset bioinformatics analysis strategy including a pathway-driven and gene-driven approach (see methods and supplementary methods; Fig. 1) to ensure that both enriched pathways as well as robust genes not annotated to pathways were maintained in the final signature (see methods). The pathway-enrichment-driven approach yielded 493 genes, the gene-driven approach 602 genes, giving a final acute asthma signature list of 933 genes.

**Figure 2:**
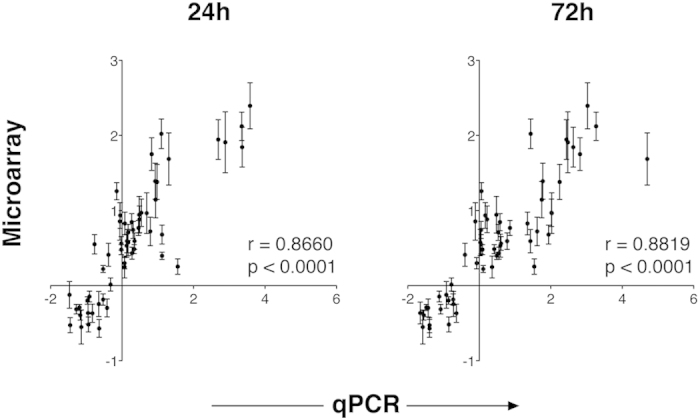
Microarray data for 59 randomly selected genes from 10 comparisons of control and asthmatic mice correlate to quantitative PCR data from our independent mouse asthma model. For qPCR, OVA-sensitized BALB/c mice received PBS (controls) or OVA challenge 24 h or 72 h before extraction of total lung RNA. Data are shown as mean ± SEM. Pearson r coefficients and p values for each correlation are indicated.

We validated the robustness of the signature by measuring 59 genes through qPCR in an independent experimental model of allergic asthma (Supplementary Fig. S1). Despite the difference in experimental setup and technology platform, we observed a significant positive correlation of 0.8660 and 0.8819 (both *p* < 0.00001) between the two datasets at 24 h and 72 h post-challenge, respectively (Fig. 2, Supplementary Fig. S3). These data indicate that stringent and multi-dataset bioinformatics analysis across independent experiments can yield robust gene signatures, which are found to be applicable regardless of experimental context and are representative of general features of the disease.

### Allergic asthma ignorome

**Figure 3:**
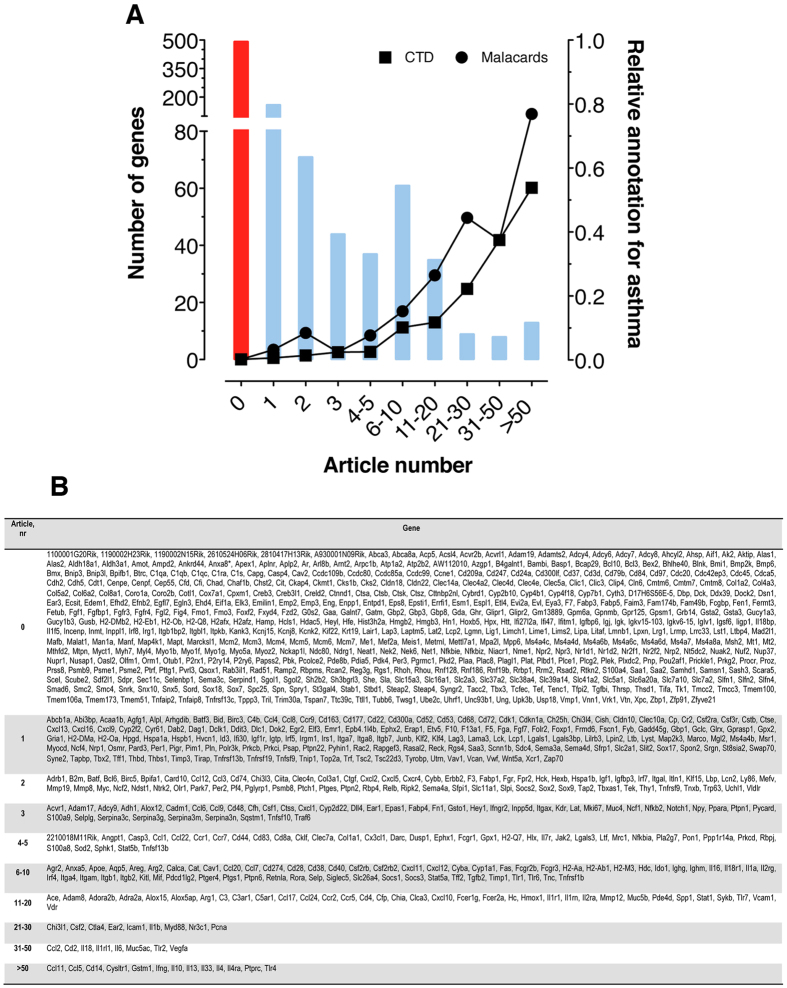
An acute asthma ignorome. (**A**) PubMed literature coverage of 933 genes from acute asthma signature list related to “asthma”. The *x*-axis represents the number of asthma-annotated literature for each gene in PubMed in November 2014. The left *y*-axis shows the gene number (shown as bars), while the right *y*-axis indicates the relative annotation to gene-disease association databases (Comparative Toxicogenomics Database (CTD) or Malacards; shown as lines). (**B**) List of MGI gene symbols for 933 asthma signature genes according to their number of asthma-annotated literature in PubMed according to the method described in supplementary methods and exact number of publications is listed in Supplementary Table 2.

To determine the already known association of the 933 genes with allergic asthma, we enumerated publications associated with asthma, as well as asthma annotation in other sources (CTD and Malacards, see supplementary methods). Figure 3A illustrates the number of genes associated with asthma using these methods. There is an inverse correlation between these data sources and the number of genes, i.e. a large number of publications are associated with relatively few genes: 13 genes (*Ccl11*, *Ccl5*, *Cd14*, *Cysltr1*, *Gstm1*, *Ifng*, *Il10*, *Il13*, *Il33*, *Il4*, *Il4ra*, *Tlr2*, and *Tlr4*) account for approximately 1/3 of the articles published on asthma for these 933 genes (Fig. 3). In our dataset, there are 493 genes (Fig. 3B) that are as yet unrelated to asthma and belong to the acute allergic asthma “ignorome”. These data, combined with the robustness of the gene signature shown above, substantiate that the scientific community has so far focused only on a small subset of genes which are likely to play a role in asthma and that there is a substantial set of genes which warrant further investigation.

### Network connectivity analysis

**Figure 4:**
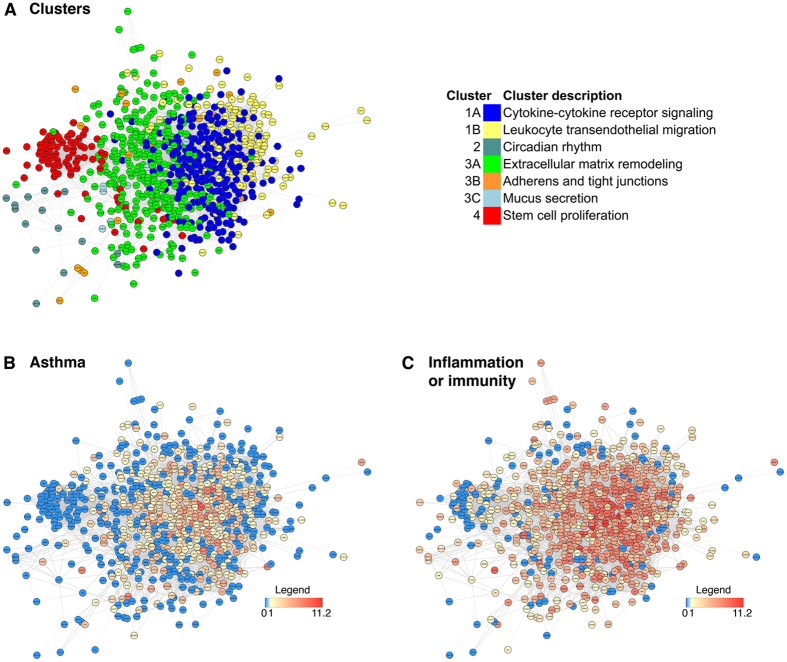
Network representation of 759 human ortholog genes in 7 clusters from our asthma-signature list. (**A**) Within the network, we have detected 7 main clusters using STRINGdb library in Bioconductor that were further functionally annotated to reveal biological functions with online tool EnrichR (for details please refer to supplementary material). Genes are coloured according to literature number associating each gene to (**B**) “asthma” or (**C**) “inflammation OR immunity”. For each gene, we have retrieved number of publications in PubMed in November 2014. Organic layout algorithm was used to produce this figure.

To define the allergic asthma gene network, we analysed the connections of 897 human orthologs from 933 acute asthma mouse genes (see methods). We detected a network of 779 densely connected genes in which there are 7 topological clusters containing 763 genes (Fig. 4A), whereas 118 genes were outside of the network (Supplementary Table S1). In the 7 topological clusters, 409 genes are associated with asthma publications (Fig. 4B) and 645 are related to “inflammation or immunity” (Fig. 4C) in at least one relevant publication.

The 7 topological clusters were annotated for functional enrichments (see methods) (Table 2, Supplementary Fig. S4). The clusters fall into 4 major biological domains, of which two are well studied, and two novel: 1) inflammation, including clusters 1A,B: cytokine-cytokine receptor signaling, leucocyte endothelial migration (353 genes, 66.86% published in asthma) 2) circadian rhythm, cluster 2, including genes expressed in the lung as well as CD4^+^, CD8^+^, CD56^+^ and CD71^+^ cells (15 genes, 86.7% as yet unpublished in asthma), 3) lung-specific insult response including clusters 3A–C: extracellular matrix remodelling, adherens and tight junctions, and mucus secretion (309 genes, of which 48.5% are published in connection to asthma), and 4) stem cell proliferation in CD34^+^, CD71^+^, CD105^+^ cells (82 genes, of which 85.4% unpublished in asthma) (Table 2, Fig. 4A).

**Figure 5:**
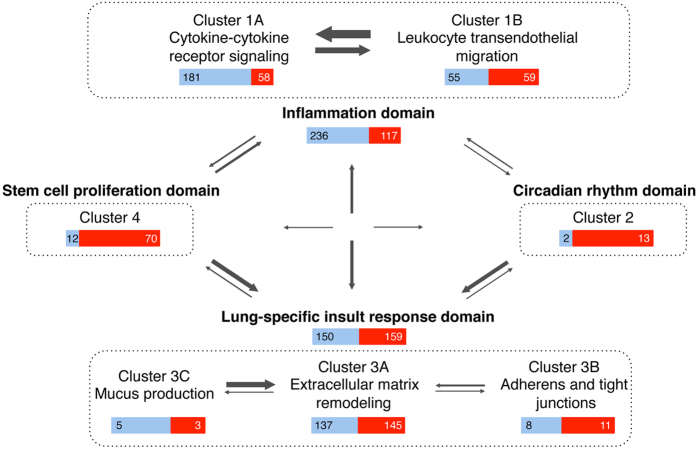
Schematic diagram depicting connectivity between 4 domains and individual clusters in acute asthma signature list. Bars indicate number of asthma-related (blue) and –ignorome (red) genes in each cluster or domain. Arrows indicating connections between clusters or domains are scaled according to relative connection strength between clusters and domains (see supplementary methods for details).

Investigating, in greater depth, the connectivity between the genes contained in these domains (see supplemental methods) enabled the characterization of the connectivity hierarchy amongst them (Fig. 5). As expected, clusters belonging to the inflammation domain have the strongest connectivity between themselves, as well as lung to inflammation domains. Interestingly, the genes within the 2 novel domains identified (stem cell proliferation and circadian rhythm) are strongly connected to a specific cluster within the lung-specific insult response, *i.e.* extracellular matrix remodelling. The same domains are also interconnected by a single gene, *Rorα* that belongs to the circadian rhythm domain and couples the stem cell proliferation and the extracellular matrix remodelling cluster in the lung-specific insult response domain (Fig. 5).

When these novel clusters are annotated for publications in inflammation or immunity, they reveal a substantially larger body of literature (73.3% of circadian rhythm genes and 56.1% of the stem cell genes are associated), indicating that their role in the broader field of immunology is already being investigated, although not yet associated specifically to asthma.

The reliability of the identified functional gene clusters is corroborated by manual inspection of well established key genes in asthma, *e.g.,* the inflammation cluster contains lung allergic immune response genes (*e.g.,* cluster 1A: *Il4, Il4r, Il13, Il33, Ccl11, Ccl17* and *Ccl22*; and cluster 1B: *Cysltr1*, *Alox5ap*, *Arg1*, *Adam8* and *Fcer1g*), the lung-specific insult response clusters with known extracellular matrix remodelling, tight junctions, and mucus production genes (*e.g., Gstm1, Vegfa, Chi3l1, and Pcna,* in cluster 3B, *e.g., Pard3*, *Prkci*, and in cluster 3C, *e.g., Muc4, Muc5b and Muc5ac*). In contrast, stem cell proliferation clusters contain genes that are essential for DNA replication and cell proliferation, which were not previously attributed to asthma. Enrichment analysis indicated that these genes are expressed mainly in cells that mediate leukocyte proliferation [CD34^+^ and CD71^+^ cells^21^,^22^,^23^] and tissue regeneration [CD105^+^ cells^24^] in allergic asthma. Cluster 2 contains well-known circadian rhythm genes *e.g., Nr1d1* (or *Rev-Erbα*), *Nr1d2*, *Per2*, and *Per3.* Most of them have not been related to asthma yet, but growing evidence suggests they play a role in the immune system^25^,^26^. This evidence suggests that our approach is able to identify valid functional clusters within the overall acute asthma gene signature and to identify genes that are less explored in the context of asthma.

We utilized the network connectivity information to prioritize specific genes that are likely to play an important role in the above domains. We concentrated on two categories of genes: hub genes, *i.e.* genes that are central, given the network structure and peripheral genes, *i.e.* genes which, despite not having a large number of connections themselves, are connected to a set of genes having full mutual interplay (*cliques*, see methods). The assumption is that hub genes play an important role in the overall network, while peripheral genes have reciprocal influence on specific *cliques*. This approach led to the identification of 34 hub genes and 8 peripheral genes in the inflammation domain, and 29 hub genes and 11 peripheral genes in the lung-specific insult response domain. The majority of the peripheral genes (14/22) have not yet been associated with asthma, but most of them have been associated with inflammation (17/22; *p* = 0.0139 vs. asthma-associated peripheral genes, Fisher’s exact test). In contrast, only 11/61 hub genes have not been associated with asthma (*p* = 0.0002 vs. asthma-associated peripheral genes, Fisher’s exact test), and 4/61 have not been associated with inflammation (*p* = 0.051 vs. inflammation-associated peripheral genes, Fisher’s exact test). Interestingly, some well-studied genes are hubs, e.g., as *Ifng, Tlr4, and Ptprc*, but several others are not, e.g., *Il13, Il4,* and *Il4r*. The ignorome hubs and peripheral genes are summarized in Supplementary Table S2. They include *Igsf6* and *Slc15a3*, which are expressed on antigen presenting cells and involved in antigen recognition^27^,^28^, as well as the peripheral gene *Clec5a* (also known as *Mdl-1*), which is involved in the innate immune response to microorganisms^29^,^30^,^31^. Within the lung-specific insult response domain, a hub gene *Tgfbi* and a peripheral gene *Bambi* belong to the *Tgfß* signalling pathway, important for lung remodelling^32^.

### Super-connector genes link functional domains

**Figure 6:**
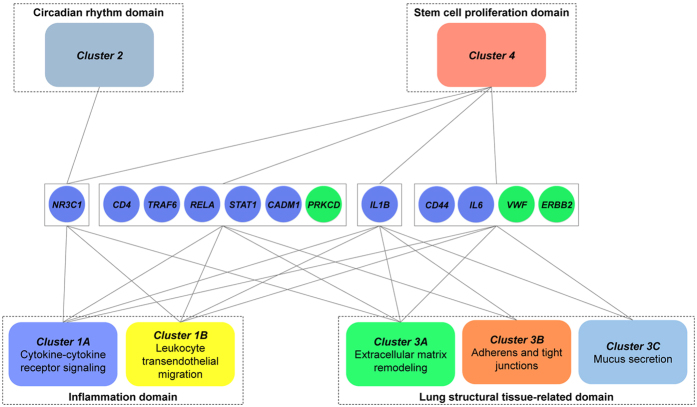
Schematic diagram depicting 12 super-connector genes in the asthma-signature gene list. Gene circle colour indicates the cluster of origin and connected to the pertaining topological cluster. Topological clusters are grouped into 4 biological domains by dotted rectangles.

We then searched for “super-connectors”, *i.e.* genes that link at least 5 clusters, and thus, identified 12 genes (Fig. 6). There are 9 super-connectors in the inflammation domain that are cytokines *Il6* and *Il1ß*, cell surface receptors *Cd4* and *Cd44*, signaling molecules *Stat1*, *Traf6,* and *Rela*, as well as *Cadm1*, a receptor that mediates mast cell adhesion to lung structural cells^33^ and the glucocorticoid receptor *Nr3c1*. The other 3 of 12 molecules belong to cluster 3 (extracellular matrix remodelling) and include *Prkcd*, a protein kinase that plays an important role in tissue remodelling^34^, an anti-haemophilic factor *Vwf* with multiple pro-inflammatory roles^35^, and *Erbb2*, an orphan tyrosine kinase that plays an important role in cancer^36^).

**Figure 7:**
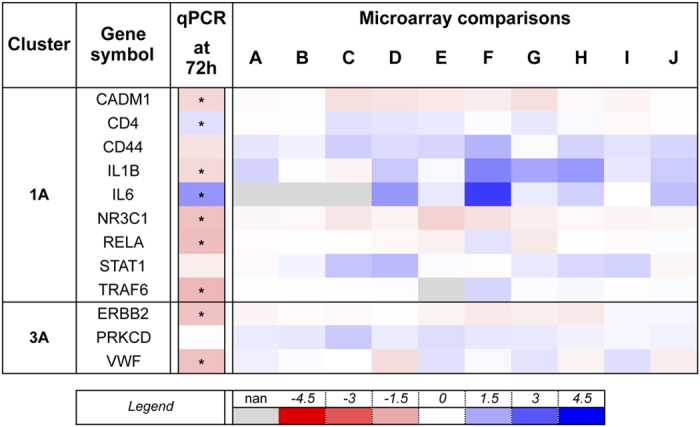
Expression profiles of 8 from 12 super-connectors were confirmed with quantitative PCR. Data are presented as mean log_2_ fold changes of gene expression by quantitative real-time PCR and microarray relative to control mice. Quantitative real-time PCR data were determined in whole lung extracts and are pooled from 2 independent experiments (n = 6). Microarray data are from 6 publicly available datasets broken down into 10 direct comparisons of asthmatic and control mice (please refer to Supplementary data and Fig. 1 for details). *p < 0.05 compared with PBS challenged mice (unpaired *t*-test).

The super-connector fold change fluctuated considerably, albeit in limited ranges, as indicated by the median fold changes that ranged from −0.01 and 0.32 within each microarray comparison (Fig. 7). This finding is consistent with a previous analysis^37^, indicating that genes with high connectivity have minimal fold change in gene expression. Despite this, we found that the expression change determined by qPCR was significant for 9/12 genes and concordant with the microarray data for 8 of 12 (67%) super-connector genes (Fig. 7). Moreover, we observed a significant positive correlation of 0.69 (*p* = 0.0132) between the two datasets at 72 h post-challenge (Supplementary Fig. S5). These data indicate that genes, which are likely to play an important role, would often escape attention in a single experiment analysis due to their minimal fold-change, but can emerge from an approach that combines computational dataset analysis and gene network connectivity investigation.

### Super-connectors, hubs and peripheral genes are affected by dexamethasone (DEX) treatment

**Figure 8:**
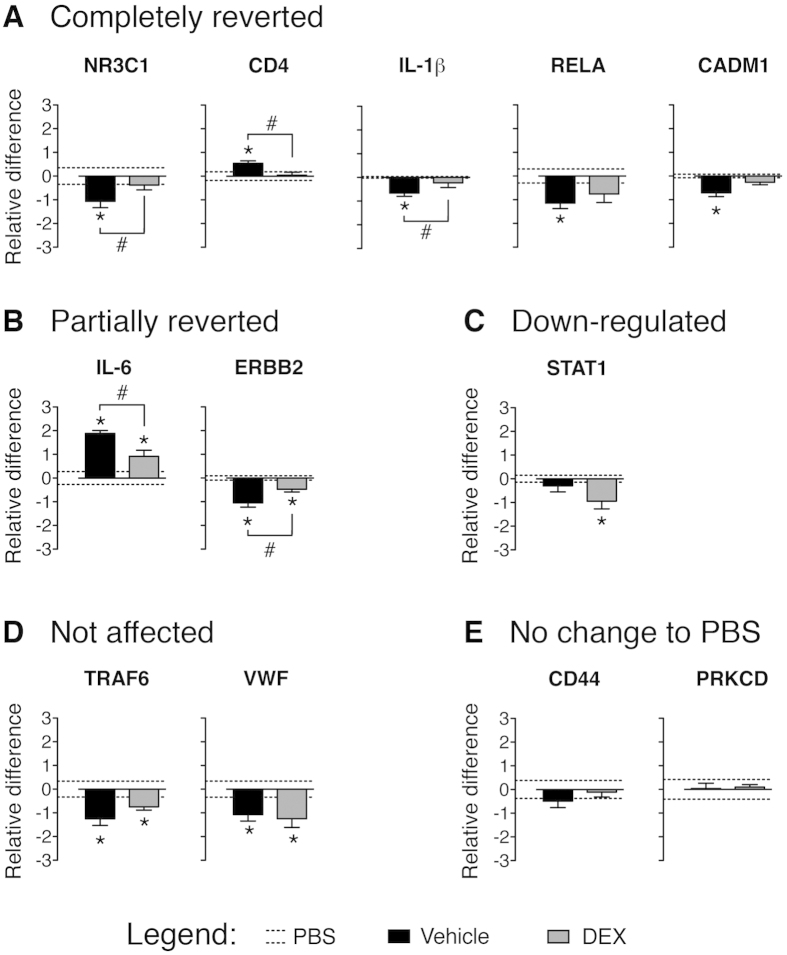
Expression of super-connectors in acute allergic asthma after DEX treatment. Total lung RNA was extracted from mice intranasally challenged with PBS (controls) and mice that received vehicle or DEX before and after OVA challenge to induce allergic asthma. Samples were collected at 72 h after allergen challenge and super-connector expression was determined with quantitative PCR. Super-connectors are grouped according to their change to DEX treatment into (**A**) completely reverted, (**B**) partially reverted, (**C**) further down regulated, (**D**) not affected with DEX treatment, and (**E**) not changed in comparison to PBS controls. Data are presented as mean ± SEM log_2_ fold changes of gene expression relative to control (PBS) mice and are pooled from 2 independent experiments (n = 6). *p < 0.05 compared with PBS challenged mice; ^#^*p* < 0.05 compared with vehicle-treated group (unpaired *t*-test).

The glucocorticoid receptor *Nr3c1* is the only super-connector that linked all 4 major domains and robustly changed expression across microarray comparisons and our qPCR. Given existing knowledge of the role of *Nr3c1* in allergic asthma, we tested the effect of DEX, a steroid that binds to Nr3c1^38^, in our animal model on both the disease phenotype and super-connector gene expression. As expected, DEX significantly suppressed airway and lung inflammation, mucus hypersecretion and lung Th2 cytokine gene expression (Supplementary Fig. S6). Out of the 9 super-connector genes that were significantly deregulated in our model, DEX partially or completely reverted 7 genes towards control values (Fig. 8). Although *Stat1* was not deregulated in our model, it was down-regulated significantly after DEX treatment, which is in agreement with a previous study^39^. *Traf6* and *Vwf* were the only 2 super-connectors significantly down-regulated in our model that were unaffected by DEX treatment. Out of the 9 super-connectors belonging to the inflammation domain, 6 were partially or completely reverted by DEX treatment, whereas of the 3 super-connectors belonging to extracellular matrix remodelling, *Erbb2* was partially reverted by DEX, *Prkcd* was not deregulated, and *Vwf* was not reverted by the treatment. To determine whether genes other than super-connectors, were affected by DEX treatment, we randomly selected 12 hub genes and 4 peripheral genes from 6 large clusters in addition to the *Muc5ac* gene as a representative gene in cluster 3C, because this cluster does not contain peripheral or hub genes. We found that 15 of 17 analyzed genes (10 hub genes, 4 peripheral genes and *Muc5ac*) were differently regulated in our model and only one (hub gene *Ifng*) was not fully or partially reverted by DEX treatment (Supplementary Fig. S7). These data indicate that systemic treatment with glucocorticoids modulates genes regardless of their topological properties in the network.

## Discussion

Here, we implement a novel computational approach used to analyse multiple microarray datasets derived from lungs of mice with acute onset allergic asthma and integrate it with independent experiments to unravel the asthma ignorome and dissect it into domains, clusters, hubs and peripheral genes.

Technical limitations reduce the power of microarrays in detection of potentially important genes with limited variation in expression levels, especially when the sample size is small^40^,^41^. Gene signals from small specific cell populations in complex tissues, such as lung, are hence diluted to low levels that are not accurately detected by microarray analyses^40^,^41^. Diverse experimental protocols that use different allergens, mouse strains, sampling times may generate gene lists that reflect differences in the kinetics of the underlying biology of the allergic asthma response. Merging individual gene lists from experiments with varying conditions and different microarray platforms may exclude key experimental condition-specific genes or pathways. To address this issue, we utilized a pathway-driven approach, which only considers genes associated with significantly enriched GO.BP terms. The novelty of our overall strategy is the use of this pathway-driven approach, and, more importantly, the combination of both pathway- and gene-driven approaches. Our strategy reduced the biological noise and detected genes with significant <1.5-fold changes that were excluded from the previous individual microarray studies^2^,^10^,^19^,^20^ and meta-analysis^16^.

Our signature gene list consists of 41% asthma-annotated genes and 59% of genes not previously associated with disease, defined as the ignorome^17^. Of the asthma-annotated genes, 13 genes associated with Th2 and inflammatory responses account for 1/3 of all asthma related publications and the remaining genes play roles in inflammation. Strikingly, there are a large number of ignorome genes involved in inflammatory pathways, DNA replication, cell cycle and wound healing, which may include important disease targets. Taken together, these data support the notion that biomedical research is skewed towards a relatively small number of ‘successful’ genes^12^,^13^,^17^,^42^. We argue that novel insights into the pathogenesis of allergic asthma should be achieved irrespective of the current literature bias. The main ignorome clusters relate to stem cell proliferation and circadian rhythm domains. Both functional domains are well established in asthma^21^,^22^,^24^,^43^ and yet the individual genes identified in this study have as yet not been associated to it, thus providing interesting candidates to pursue these functional areas in the disease.

Understanding hubs (predominantly asthma-annotated) and peripheral genes (mostly ignorome) is essential for establishing functional interplay within the clusters and determining why key Th2 genes e.g., *Il13, Il4,* and *Il4r* are neither hub nor peripheral genes^44^,^45^. These data support the finding that there is no correlation between a gene’s degree of interaction, importance in the pathogenesis, and frequency of occurrence in the scientific literature^14^.

Notably, all super-connectors are asthma-annotated and belong to either inflammation or lung-specific insult response domains. In the DEX experiment, we found that only *Traf6* and *Vwf* had not changed expression levels. However, it is possible that they might have changed, if higher steroid doses were used, especially because steroids are known to increase *Vwf*^46^,^47^ and *Traf6*^48^ expression. Although we focused on these super-connectors, there is evidence that 41% (314) of genes interconnect 3 or more clusters, 1/3 of them being an ignorome, further demonstrating a large constellation of interrelated clusters across tissues and biological domains.

There are caveats regarding the signature gene list and analyses presented here. Firstly, these analyses are based on the early events at the initiation of acute allergic asthma in the lungs of mice and may not reflect the same multiplicity of clusters and biological domains that would appear when testing chronic disease or disease relapse. Secondly, according to the current paradigm, genes in the inflammatory and immune response are expected, however, the remodelling response appears earlier than expected and these genes may reflect an early response of the lung to inflammatory signals and may represent the activation of healing mechanisms. Thirdly, it is not known whether human gene expression profiles from patient lungs would produce a similar gene list and functional clusters, because there are only 2 publically available human microarray datasets on lung biopsy samples [GEO database (http://www.ncbi.nlm.nih.gov/geo/)]. Importantly, this approach would enable comparisons between human and mouse datasets providing excellent opportunities to validate experimental models.

Asthma is a heterogeneous disease that includes several distinct disease phenotypes and endotypes^49^ and is not usually studied at acute onset, but rather during ongoing disease. Multiple dataset analyses including more gene expression profiles from mouse and man and at different stages of disease and in distinct patient subgroups may result in the prioritization of essential genes and networks at the core of disease and simultaneously highlight molecular differences between phenotypes.

In conclusion, the integration of prior literature knowledge and unbiased multiple dataset analysis paradigms through the lens of systems biology provides important insights into asthma pathogenesis, allowing to bring novel biology and existing knowledge on a balanced plate, highlighting connections between the two and providing novel avenues to combat the disease.

## Methods

### Data selection

From the 23 experiments identified in the literature, we selected 6 datasets based on studies using the Affymetrix Mouse Genome 430 2.0 or Murine Genome U74 Version 2 Arrays microarray platform and mouse models of ovalbumin (OVA) and house dust mite induced acute asthma characterized by airway eosinophilia, airway hyperresponsiveness (AHR), and mucus hypersecretion. Within these independent datasets, we extracted in total 10 comparisons of asthmatic vs. control samples (see Table 1).

### Multi-dataset bioinformatics analysis for selection of acute asthma signature list

We retrieved raw data from the GEO database^1^ and analysed them using a common pipeline for signal normalization and extraction of differentially expressed genes (see supplementary methods and Fig. 1). We extracted differentially regulated genes for each experiment, which were then utilized for our multi-dataset bioinformatics analysis combining two approaches: a top-down, biological process enrichment-driven approach, from now on called ‘pathway-driven’, and a bottom-up, ‘gene-driven’ approach (see supplementary methods). The two approaches were merged to generate a final union list.

### Gene annotation for acute asthma ignorome detection and its functional annotation

To assess the extent of the acute asthma ignorome, we performed an unambiguous literature search based on Entrez Gene and PubMed databases (see supplementary methods). To annotate the genes from the acute asthma signature list (including the asthma ignorome), we compiled a list of human orthologs, mapped it to the STRING interaction network, and performed a topological analysis for clusters of densely connected genes (*n* ≥ 5; details in supplementary methods). For each cluster, we identified three sets of genes: (i) hub genes (the top 10% of the genes in the list ranked by betweenness centrality, defined as the ratio of shortest paths passing through a node, being a common measure of centrality of a node parameter in descending order); (ii) peripheral genes (1-degree nodes connected to a clique, *i.e.* a set of nodes completely connected to each other); and (iii) super-connectors (genes connecting more than 5 clusters). Each cluster was then further characterized in terms of functional enrichment, tissue specific expression and literature representation (see supplementary methods).

### Biological validation experiments

We used 8–10 week old female BALB/c mice (Charles River, Sulzfeld, Germany). All experimental protocols were approved by the ethical committee of the Medical University of Vienna and the Animal Care Committee of the Austrian Ministry of Science and carried out in accordance with the approved guidelines. All mice were immunized with OVA (Sigma Chemical Co., St. Louis, MO) dissolved in PBS intraperitoneally on days 0 and 21 and intranasally on day 32 with OVA or PBS. In treatment experiments, we administered vehicle or DEX (Sigma) intraperitoneally at 1 mg/kg 30 min before and 24 and 48 h after challenge. Details on animal treatment and measurements of airway hyperresponsiveness, airway and lung inflammation, mucus hypersecretion and serum OVA-specific antibody titers are reported in supplementary methods. Separate groups of mice were used for lung mRNA extraction at 24 or 72 h after intranasal challenge with PBS or OVA. Methods for RNA extraction, reverse-transcription, quantitative polymerase chain reaction and statistical analysis are reported in supplementary information.

## Additional Information

**How to cite this article**: Riba, M. *et al.* Revealing the acute asthma ignorome: characterization and validation of uninvestigated gene networks. *Sci. Rep.* **6**, 24647; doi: 10.1038/srep24647 (2016).

## Supplementary Material

Supplementary Information

Supplementary Table S1

## Figures and Tables

**Table 1:** Gene expression studies of asthma used for the bioinformatics analysis.

No.	Study				Affimerix Mouse Array	Mice	Antigen (Mice number)	No. of challenges	Sampling time (hr after last challenge)
	GSE	GPL	PMID	Comparison					
1	13032	1261	19491150	A	Genome 430 2.0	A/J	PBS (3)	3	3
							OVA (3)		
				B		B6	PBS (3)	3	3
							OVA (3)		
2	6858	1261	17437023	C	Genome 430 2.0	BALB/c	PBS (4)	7	24
							OVA (4)		
3	9465	1261	19057703	D	Genome 430 2.0	A/J	PBS (3)	1	96
							OVA (3)		
4	1301	339–340	–	E	U74 Version 2	BALB/c	PBS (3)	2	72
							HDM (3)		
5	3184	339	–	F	U74 Version 2	C3H	PBS (5)	1	6
							OVA (5)		
				G		C3H	PBS (5)	1	24
							OVA (5)		
				H		A/J	PBS (5)	1	6
							OVA (5)		
				I		A/J	PBS (5)	1	24
							OVA (5)		
6	18010	1261	19770271	J	Genome 430 2.0	B6.Il4raQ576/Q576	-(5)	7	24
						B6.Il4raQ576/Q576/IL-13tg	IL-13 (8)		

Six GEO Datasets (http://www.ncbi.nlm.nih.gov/gds/) studies were selected for meta-analysis on the basis of the microarray platform and *in vivo* experimental protocol. From those 6 studies, 10 asthmatic *vs.* healthy control comparisons (A to J) have been extracted and further analyzed.

**Table 2:** Biological processes, pathways and tissue expression enrichment analysis of main clusters in asthma-signature genes list, examples of hubs, peripheral genes, super-connectors and genes present in Malacards, a gene-disease association database.

Cluster		1A	1B	2	3A	3B	3C	4
Description		Cytokine-cytokine receptor siganling	Leukocyte transendothelial migration	Circadian rhythm	Extracellular matrix remodeling	Adherens and tight junctions	Mucus secretion	Stem cell proliferation
Gene number		239	114	15	282	19	8	82
Gene Ontology Biological Processes^1^		Immune system process (GO:0002376); Defense response (GO:0006952); Immune response (GO:0006955); Chemotaxis (GO:0006935); Response to external stimulus (GO:0009605)	Immune system process (GO:0002376); Defense response (GO:0006952); Immune response (GO:0006955); Innate immune response (GO:0045087); Respiratory burst (GO:0045730)	Regulation of RNA metabolic process (GO:0051252); Regulation of transcription, DNA-dependent (GO:0006355), Heme biosynthetic process (GO:0006783), Regulation of transcription from RNA polymerase II promoter (GO:0006357), Regulation of transcription (GO:0045449)	Regulation of cell proliferation (GO:0042127); Negative regulation of apoptosis (GO:0043066); Negative regulation of programmed cell death (GO:0043069); Regulation of cell migration (GO:0030334); Regulation of cell motion (GO:0051270)	Establishment or maintenance of cell polarity (GO:0007163)	Monosaccharide metabolic process (GO:0005996); Cellular alcohol metabolic process (GO:0006066); Ventricular cardiac muscle morphogenesis (GO:0055010); Cardiac muscle contraction (GO:0060048); Carbohydrate metabolic process (GO:0005975)	Organelle organization (GO:0006996); Chromosome organization (GO:0051276); Regulation of cell cycle (GO:0051726); DNA replication (GO:0006260); DNA metabolic process (GO:0006259)
KEGG Pathways^1^		Cytokine cytokine receptor interaction (HSA04060); Hematopoietic cell lineage (HSA04640); Toll like receptor signaling pathway (HSA04620); Cell adhesion molecules (HSA04514); JAK STAT signaling pathway (HSA04630)	Leukocyte transendothelial migration (HSA04670); Fc epsilon ri signaling pathway (HSA04664); Phosphatidylinositol signaling system (HSA04070); Natural killer cell mediated cytotoxicity (HSA04650); Inositol phosphate metabolism (HSA00562)	Circadian rhythm (HSA04710); Glycine serine and threonine metabolism (HSA00260); Arginine and proline metabolism (HSA00330); Porphyrin and chlorophyll metabolism (HSA00860)	ECM receptor interaction (HSA04512); Focal adhesion (HSA04510); Complement and coagulation cascades (HSA04610); p53 signaling pathway (HSA04115); Arachidonic acid metabolism (HSA00590)	Tight junction (HSA04530)	Keratan sulfate biosynthesis (HSA00533); Glycan structures biosynthesis 1 (HSA01030); Olfactory transduction (HSA04740); Galactose metabolism (HSA00052)	Cell cycle (HSA04110); Purine metabolism (HSA00230); Pyrimidine metabolism (HSA00240)
Human Atlas Enriched Tissues2,^3^		CD33^+^ Myeloid cells CD19^+^ B cells CD14^+^ Monocytes Whole blood Smooth muscle	CD14^+^ Monocytes CD33^+^ Myeloid cells Whole blood Lung	CD71^+^ early erythoid cells Lung CD56^+^ NK cells CD8^+^ T cells CD4^+^ T cells	Smooth muscle Lung	Bronchial epithelial cells CD105^+^ Endothelial cells CD34^+^ cells Whole blood CD71^+^ early erythoid cells	Trachea CD71^+^ early erythoid cells CD56^+^ NK cells CD14^+^ Monocytes Lung	B lymphocytes CD105^+^ Endothelial cells CD71^+^ early erythoid cells CD34^+^ cells
Selected genes	Hubs^4^	*Cd4, Il6, Stat1, Ifng, Cxcl10*	*Tyrobp, Plek, Itgb2, C1qa, Slc15a3*	*Alas1, Dbp, Gatm, Alas2, Nr1d1*	*Vegfa, Fn1, Erbb2, Itgb1, Cav1*	*Prkci, Sqstm1, Pard3, Pln, Gria1*	*Muc5b, St3gal4, Clca1, Gaa, Tff2*	*Mad2l1, Nme1, Pttg1, TK1, Rrm2*
	Peripheral^5^	*Mtpn, Hla-dbq2*	*Cotl1, Prickle1, Siglec8, Sdpr, Spon2, Clec5a*	–	*Tfpi2, Aqp5, Bambi, Myo1b, Efhd2, Trpan7, Mmp19, Myc, Itgb1bp2, Tmem173, Bex1*	–	*Tff2, Slc26A4, Chst2*	*Adam19, Cd14*
	Super-connectors	*Nr3c1, cd4, Traf6, Rela, Stat1, Cadm1, Il1b, Cd44, Il6*	–	–	*Prckd, Vwf, Erbb2*	–	–	–
	Malacards Asthma^6^	*Il13, Ccl11, Hhla-g, Il4r, Tlr6, Rnase3, Il4, Ccl17, Ccl5, Fcer2*	*Pla2g7, Alox5ap, Cysltr1, Adam8*	–	*Chia, Alox12, Alox15, Cat*	–	–	–
Percentage of genes in PubMed related to	“asthma”	75.7%	48.2%	13.3%	48.6%	42.1%	62.5%	14.6%
	“Inflammation or immunity”	96.7%	84.2%	73.3%	84.8%	63.2%	100.%	56.1%

^1^Maximum of 5 enriched categories ordered by Enrichr Combined score showing an adjusted P-value < 0.05.

^2^Maximum of 5 tissues within a list of 17 tissues potentially found in the whole lung tissue samples in datasets under study (See

^3^Supplementary Figure S4 for details on Human Atlas Gene expression for all genes in each cluster).

^4^Top 5 hub genes ordered by Betweenness Centrality.

^5^Peripheral genes defined as 1-degree nodes connected to a clique.

^6^Malacards overlap for Asthma (119 genes, as for Sept 2014).
