# Supplementary material for: Revealing the acute asthma ignorome: characterization and validation of uninvestigated gene networks
Source: Sci Rep. 2016 Apr 21;6:24647. doi: 10.1038/srep24647 (PMC4838989; doi:10.1038/srep24647)
Supplement: Supplementary Information [file srep24647-s1.doc]

Revealing the acute asthma ignorome: characterization and validation of uninvestigated gene networks

Michela Riba, Jose Manuel Garcia Manteiga, Berislav Bošnjak, Davide Cittaro, Pavol Mikolka, Connie Le, Michelle M. Epstein and Elia Stupka

Online Data Supplement

Supplementary materials and methods

Supplementary tables

Supplementary figures

Supplementary references

**Supplementary materials and methods**

*Multi-dataset bioinformatics analysis*

To overcome the limitations of direct comparisons of the results obtained by multiple laboratories using differrnt analysis pipelines, we retrieved the raw data from the GEO database ^1^ and re-analyzed them using a common pipeline for signal normalization and extraction of differentially expressed genes with limma and Affy Bioconductor libraries ^2^. Raw data were normalized using a robust multi-array average [RMA; ^3^]. In each experiment, genes with expression values lower than the first quartile of the entire distribution of values were considered as non-expressed and removed from the analysis. We applied parametric moderated *t*-statistics, implemented in the limma library, to identify differentially expressed genes between asthmatic and control samples. Each differential expression analysis was conducted separately for each experiment. Significantly up- and down-regulated genes were selected using an adjusted *p*-value threshold of 0.05 (Benjamini-Hochberg correction ^4^).

The resulting gene lists were then utilized for our multi-dataset bioinformatics analyses. We combined 1) top-down, biological process enrichment-driven (pathway-driven) and 2) bottom-up (gene-driven) strategies. The pathway-driven strategy was based on the selection of common asthma-specific pathways from the combination of enriched biological terms found across individual studies. In the gene-driven strategy, a list of differentially expressed genes was created across all individual datasets, normalizing the size of the list to balance the different statistical power of each experimental design. Results from each approach were merged to generate a comprehensive list of genes. Human orthologs of the genes on the list were used to query a network of interactions and investigated in terms of topological structure using STRINGdb Bioconductor library ^5^ and Cytoscape ^6^. The details of each approach are described below.

*Pathway-driven strategy*

We performed a Gene Ontology Biological Process (GO.BP) term enrichment analysis using the lists of differentially expressed genes derived from each microarray comparison, selected at a stringency of adjusted *p*-value < 0.05, using the NCBI DAVID online tool ^7^. The 76 GO.BP were selected according to the following criteria: 1) ease score < 0.01, 2) presence in at least 2 studies, fold enrichment > 2, 3) categories which were also enriched in the background of the mouse transcriptome alone were filtered out. We calculated sematic similarity among enriched terms using the GO.SemSim Bioconductor library ^8^. The matrix of similarity scores was clustered using Euclidean distance and average linkage agglomerative method, as described previously ^9^. We observed 6 clearly visible clusters characterized by a high level of similarity. We chose to cut the hierarchical tree at 6 rather than at a higher number of clusters, because of the overlap of summary terms describing the process that would lead to a redundancy of biological process descriptors*.* The 6 major clusters of semantically related terms include, 1) regulation of morphogenesis, 2) T-cell selection, 3) antigen processing and presentation, 4) response to external stimuli, 5) immune signaling, and 6) leukocyte-mediated response (Supplementary fig. S8). Differentially expressed genes referring to enriched GO.BPs were retained to form a non-redundant “pathway-driven” list (*n*=493).

*Gene-driven strategy*

To obtain a list of differentially expressed genes (DGEs) based on a ‘gene-driven approach’, we performed a two-step analysis: normalized union of DGEs and Rank Product of DGEs.

*Normalized Union of DGEs*

The genes obtained from the limma differential expression analysis were ranked from highest to lowest significance for all 10 comparisons performed (A to J). The comparison which produced the smallest list of genes was used to define a cut-off for the remaining comparisons performed to produce 10 lists of genes of the same size that contained highly significant genes (adjusted *p*-value < 0.05). This approach was used to ensure a stringent approach for genes inclusion and to obtain a balanced contribution of each study for the downstream analyses. We then performed a non-redundant merge of these 10 lists.

*Rank Product of DGEs*

We used a non-parametric Rank Product approach to analyze the 10 experimental studies using the RankProd Bioconductor library ^10^. This approach allows the combination of diverse datasets for meta-analyses of gene expression data ^11^ by identifying up- or down-regulated genes based on consistent fold change across experiments, instead of *t* statistics. Rank Product detected genes in the comparison of asthmatic *vs.* control samples that were consistently highly ranked in several gene lists, *i.e.*, genes that were consistently strongly up- or down-regulated in replicate experiments [see ^10^ for further details]. The RankProd Bioconductor package was used to calculate rank product statistics on data from the 10 genes lists, which resulted in lists of up-regulated and down-regulated genes. These genes were then ranked based on their meta-analysis Rank Product FDR (*pfp*). We identified a *pfp* cutoff with the same number of genes from the normalized union approach, with the same up- and down-regulated proportions, to balance the contribution of both approaches. We combined the list of genes from the normalized union step (*t*-statistics) with the genes coming from the Rank Product step (non-parametric) and produced an intersection containing genes that were identified by both statistical approaches, leading to a highly stringent and robust set of differentially expressed genes which ranked highly both in terms of *t*-statistics and fold-change rank product.

*Gene Annotation*

For each gene in the list, we performed a literature search in PubMed to identify publications associated with asthma as keyword in the title or abstract. We used NCBI Entrez Gene search engine [^12^; http://www.ncbi.nlm.nih.gov/gene/] on EntrezId terms for either mouse genes or their human orthologs (see below). We used a ruby script to retrieve NCBI publication numbers using either “Asthma” or “Immunity OR Inflammation” in the title or abstract of the papers and the number of resulting publications was used to annotate each gene (date of publication number retrieval, 28^th^ of November 2014; see Supplementary Table S1). To validate 550 genes that had 0 publications for “asthma”, we performed additional searches in PubMed using the gene abbreviation as a keyword. For 444 genes, we confirmed 0 publications records for “asthma” and for the remaining 106 genes, the publication number was manually curated to retrieve a final list of 493 genes, whose function was not related to “asthma”. An overlap of our asthma signature with known as asthma-related annotated genes was calculated using the Comparative Toxicogenomics Database (CTD) via the enrichment ToppGene Suite ^13^ and with Malacards ^14^ through GeneCards [^15^, http://www.genecards.org/]. GeneCards allergic asthma-related genes were retrieved with the advanced search using “allergic asthma” as keywords in February 2014.

*Network analysis and network cluster annotation*

Pathway-driven and gene-driven lists were merged into a final union list. To address the corresponding human orthologs, we used BioMart to automatically map the human orthologs from the gene symbols ([www.biomart.org](http://www.biomart.org/)) followed by a combined search using EnsEMBL and MGI databases ([www.ensembl.org](http://www.ensembl.org/) and <http://www.informatics.jax.org/>). For mouse genes that had more than one potential human ortholog, all genes were retrieved. We used a full list of human orthologs to query STRING interaction network using STRINGdb library in Bioconductor. We performed a topological analysis using the igraph^16^ “get_clusters” function included in the STRINGdb library to search for clusters of densely connected genes, i.e., a group of nodes having high internal connectivity. When we run the get clusters function with default parameters, it returned 7 topological clusters with at least 5 genes. Clusters were scored using a *p*-value based on the probability of having more connections within themselves than with the rest of the network (see Supplementary Table S3).

We analyzed dependence of the enrichment in asthma genes changing the threshold of betweenness centrality ordered by decreasing value. We found that there is a steep increase in the enrichment when the threshold was set at the top 10% of genes (Supplementary fig. 2). Betweenness centrality is an acknowledged approach for defining hubs in a biological network ^17^. There were 304 genes, which connected more than 3 clusters, 95 genes connecting 4 clusters, and 12 genes connecting 5 clusters, there were 12 nodes, which we termed, super-connectors.

*Functional annotation of genes within 7 clusters*

For each cluster, we identified three sets of genes: hub genes, peripheral genes and super-connectors. Hubs were defined as the top 10% of the genes in the each cluster ranked by betweenness centrality parameter in descending order. Peripheral nodes were defined as 1-degree nodes connected to a clique (a maximal complete subgraph where all vertices are connected). Super-connectors were defined as genes connecting more than 5 clusters.

Each cluster was further analyzed using the online EnrichR tool ^18^. We analyzed enrichments for the human orthologs in the following gene sets: Gene Ontology Biological Process (GO.BP), KEGG Pathways, and Human_Atlas. Gene sets with *p*<0.05 were considered significant. We also extracted expression values for each gene from the BioGPS ^19^ dataset within 17 different tissues potentially represented in asthmatic lungs (<http://biogps.org/downloads/>): 12 related to leukocytes, and 5 related to lung tissues. We used the pheatmap R library [R package version 0.7.7. http://CRAN.R-project.org/package=pheatmap] to produce clustered heatmaps for genes in each cluster characterized in the network analysis (Supplementary Fig. 4).

*Calculation of relative connection strength between clusters and domains*

To assess the connectivity between different clusters as well as between biological domains, we processed the raw counts of genes and connections in each cluster and each domain. Briefly, we obtained a “connectivity ratio” for each cluster by dividing the number of connections per cluster by the square of the number of genes present in the same cluster. We used this “connectivity ratio” to normalize the number of connections of a cluster with another specific cluster by the connectivity ratio of the originating cluster. Finally, to obtain biological domain connectivity scores, we used the average of the connectivity scores of all clusters found within the biological domain.

*Acute allergic asthma and dexamethasone treatment*

In all experiments (similar to the biological validation experiments), we used 8-10 week old female BALB/c mice (Charles River, Sulzfeld, Germany) provided with OVA-free food (SSNIFF, Soest, Germany) and autoclaved water *ad libitum*. All experimental protocols were approved by the ethical committee of the Medical University of Vienna and the Animal Care Committee of the Austrian Ministry of Science and carried out in accordance with the approved guidelines. All mice were immunized with 10 μg of OVA (Sigma Chemical Co., St. Louis, MO) in 200 μl of PBS phosphate buffered saline (PBS) intraperitoneally (i.p.) on days 0 and 21 and then intranasally (i.n.) challenged on day 32 with (i) 100 μg of OVA in 50 μl of PBS or (ii) 50 μl of PBS (Supplementary Fig. 6). Dexamethasone (DEX, Sigma, 1 mg/kg) dissolved in DMSO and then diluted with PBS to a final concentration of 0.1 mg/ml (final concentration of DMSO was 5%) was administered intraperitoneal (i.p.) for a final concentration of 10 ml/kg. DEX or PBS were administered 30 min before and 24h and 48h after OVA challenge.

*Airway hyperresponsiveness*

At 24h after i.n. OVA challenge, we measured airway resistance and dynamic compliance in anesthetized and ventilated animals by Resistance and Compliance System (Buxco Electronics Ltd., Troy, NY, USA). Briefly, PBS, followed by increasing concentrations of methacholine (Sigma), was nebulized for 3 min and lung function was recorded and calculated using FinePoint software (Buxco Electronics Ltd.).

*Airway inflammation*

- Seventy-two hours after the i.n. OVA challenge, lungs were washed with total volume of 1 ml of PBS to collect bronchoalveolar lavage fluid (BAL). The total number of cells in BAL was enumerated in an improved Neubauer hemocytometer and cytocentrifuged preparations (Cytospin-4, Thermo Shandon Corporation, Pittsburg, PA, USA) were stained with the Kwik-Diff (Thermo Fisher Scientific Inc., Pittsburgh, PA, USA) to determine the differential cell count by morphological examination of ≥300 cells.

*Lung inflammation and mucus hypersecretion*

Lungs were dissected after BAL and fixed in paraformaldehyde. Paraplast-embedded lung sections (3 µm) were stained with hematoxylin and eosin (H&E) for morphological evaluation and with periodic acid-Schiff stain (PAS) for enumeration of mucopolysaccharide positive cells. The intensity and severity of inflammation on the H&E stained slides was graded according to our semi-quantitative scoring system as described previously ^20^. The number of mucus-containing cells per millimeter of basement membrane was determined with ProgRes CapturePro Software (Jenoptik, Jena, Germany).

*Serum OVA-specific immunoglobulin*

The measurement of OVA-specific IgG1 and IgE titers in sera was done by in-house ELISA as described previously ^19^.

*mRNA extraction and quantitative PCR*

Separate groups of mice were used for lung mRNA extraction at 24h or 72h after intranasal challenge with PBS or OVA. Briefly, total RNA was isolated using RNeasy Mini Kit (Qiagen, Germantown, MD, USA) and the integrity of the isolated RNA was confirmed using an Agilent 2100 Bioanalyzer and Agilent RNA 6000 Nano Assay Kit (Agilent Technologies, Pao Alto, CA, USA). After reverse-transcription with the Superscript III first-strand cDNA synthesis supermix kit (Invitrogen, Carlsbad, CA, USA), gene expression was analyzed using the SYBR green method for quantitative polymerase chain reaction (qPCR) on StepOnePlus real time PCR system (Applied Biosystems, Foster City, CA, USA). Collected data were analyzed with the ΔΔCT method for which the geometric mean of threshold cycle (CT) values for hypoxanthine-guanine phosphoribosyltransferase (HPRT), β-actin and glyceraldehyde 3-phosphate dehydrogenase (GAPDH) was used as an endogenous control and mean of CT values from recovered mice was used as a reference sample. The list of primers (Invitrogen or Microsynth, Balgach, Switzerland) can be found in Supplementary Table S4.

*Correlation of microarray and qPCR data*

Data from 59 selected genes with divergent fold changes were analyzed with qPCR and correlated to the average microarray expression across the 10 divergent microarray comparisons (A-J). To include gene into the analysis, its expression had to be detectable in control and asthmatic mice with qPCR, *i.e.* genes with a Ct value of 40 in any group were excluded from analysis.

*Statistical analysis*

To compare airway hyperresponsiveness data, area under curve (AUC) was calculated for each animal on the graph of resistance or compliance (*y* axis) vs. the methacholine concentration (mg/ml; *x* axis). Before analysis, the values for the calculated AUC for resistance and compliance, the airway inflammation data, and the mucus-positive cell counts in the lungs were log-transformed to equalize variances. Those data were analyzed with Student’s *t*-test (for experiments with control and asthmatic mice) or with one-way ANOVA followed by Tukey’s multiple comparison test (in experiments with DEX treatment). Scores for lung inflammation were analyzed with Chi-squared test for trend. A Pearson *r* correlation was used to analyze microarray and qPCR data correlation. An unpaired *t*-test was used to compare differences in gene expression determined with qPCR between control and asthmatic mice and vehicle-treated and DEX-treated OVA-challenged mice. All analyses were done using GraphPad Instat v.5.0 (GraphPad Software Inc., San Diego, CA, USA) and *p*-values were considered significant at 0.05 threshold.

**Supplementary Figure legends**


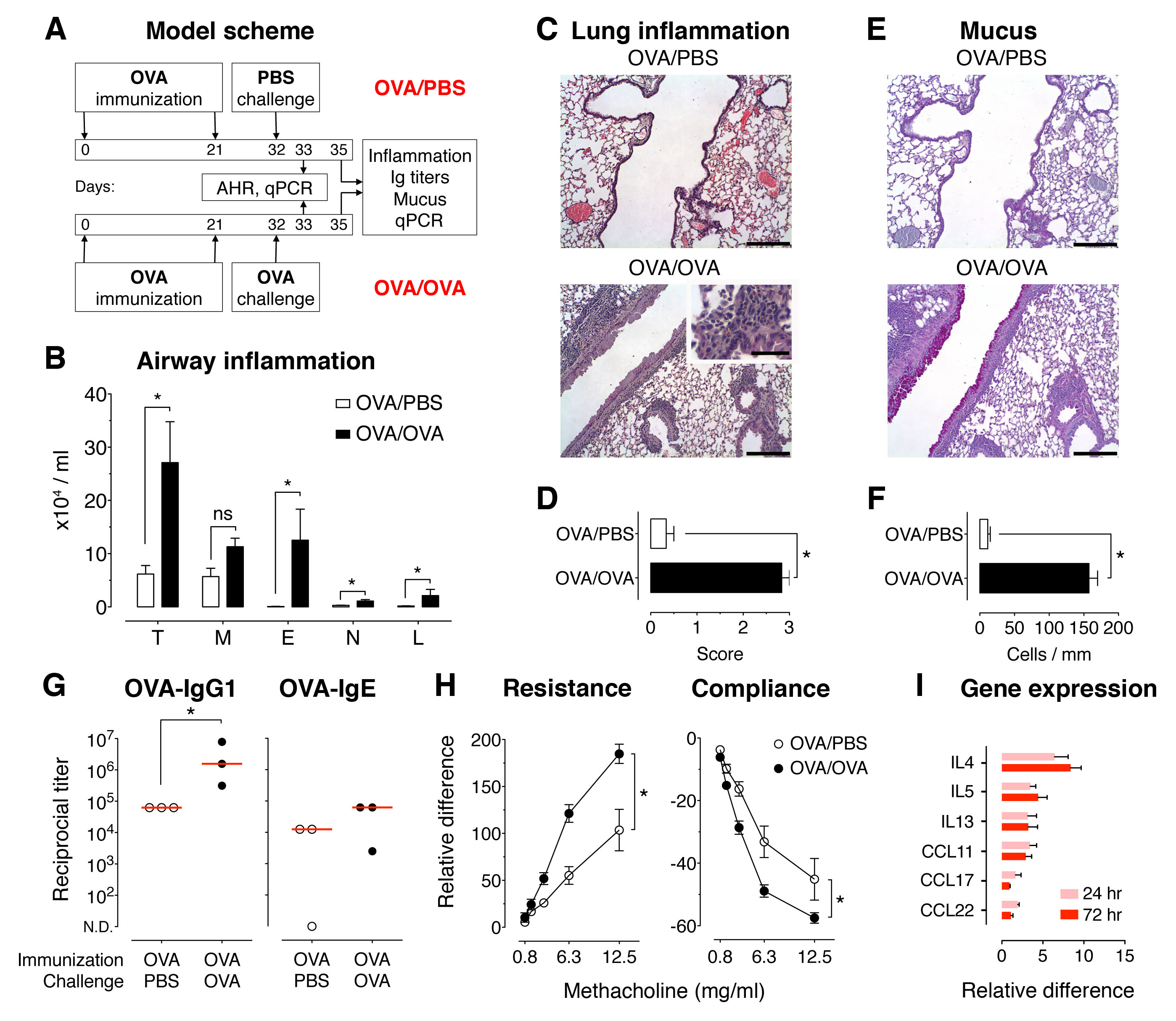


**Supplementary Figure S1.** OVA-immunized and challenged mice have increased airway and lung inflammation, mucus hypersecretion, serum allergen-specific IgG1 and IgE titers, airway hyperresponsiveness, and type-2 related cytokine and chemokine gene expression in the lungs compared to control animals. (a) Schematic representation of acute allergic asthma protocol. (b) Total and differential cell counts in BAL. T – total cells, M – macrophages, E – eosinophils, N – neutrophils, L – lymphocytes. (c) Representative photomicrographs of H&E stained lung sections. Inset shows eosinophils in inflammatory infiltrates in asthmatic mice. Scale bars: 200 µm (inset 50 µm), original objective: 10x (inset 40x). (d) Scores for lung inflammation; for description please refer to material and methods. (e) Representative photomicrographs of Periodic-acid Schiff (PAS) stained lung sections showing fuchsia-stained mucus in epithelial goblet cells of asthmatic animals. Scale bar: 200 µm; original objective: 10x. (f) Number of mucus-positive cells per millimeter of basement membrane. (g) Serum titers of OVA-specific IgG1 and IgE. (h) Airway resistance and dynamic compliance in response to methacholine is presented as the relative difference compared with the baseline values measured after PBS nebulization. (i) Increased mRNA expression of selected type-2 immune response cytokines and chemokines in whole lungs at 24h and 72h after OVA challenge. Data are expressed as the relative difference to corresponding PBS-challenged mice. Data in A-G and I are shown as mean ± SEM and are representative of at least 2 independent experiments (*n* = 3). Data in h are shown as mean ± SEM (n=5). (B-H) * *p* < 0.05 unpaired Student’s *t*-test.

**Supplementary Figure S2.** Dependence of the enrichment in asthma genes (y-axis) at different percentiles of betweenness centrality (x-axis) providing an indication for the selection of the threshold at the top 10% of genes.


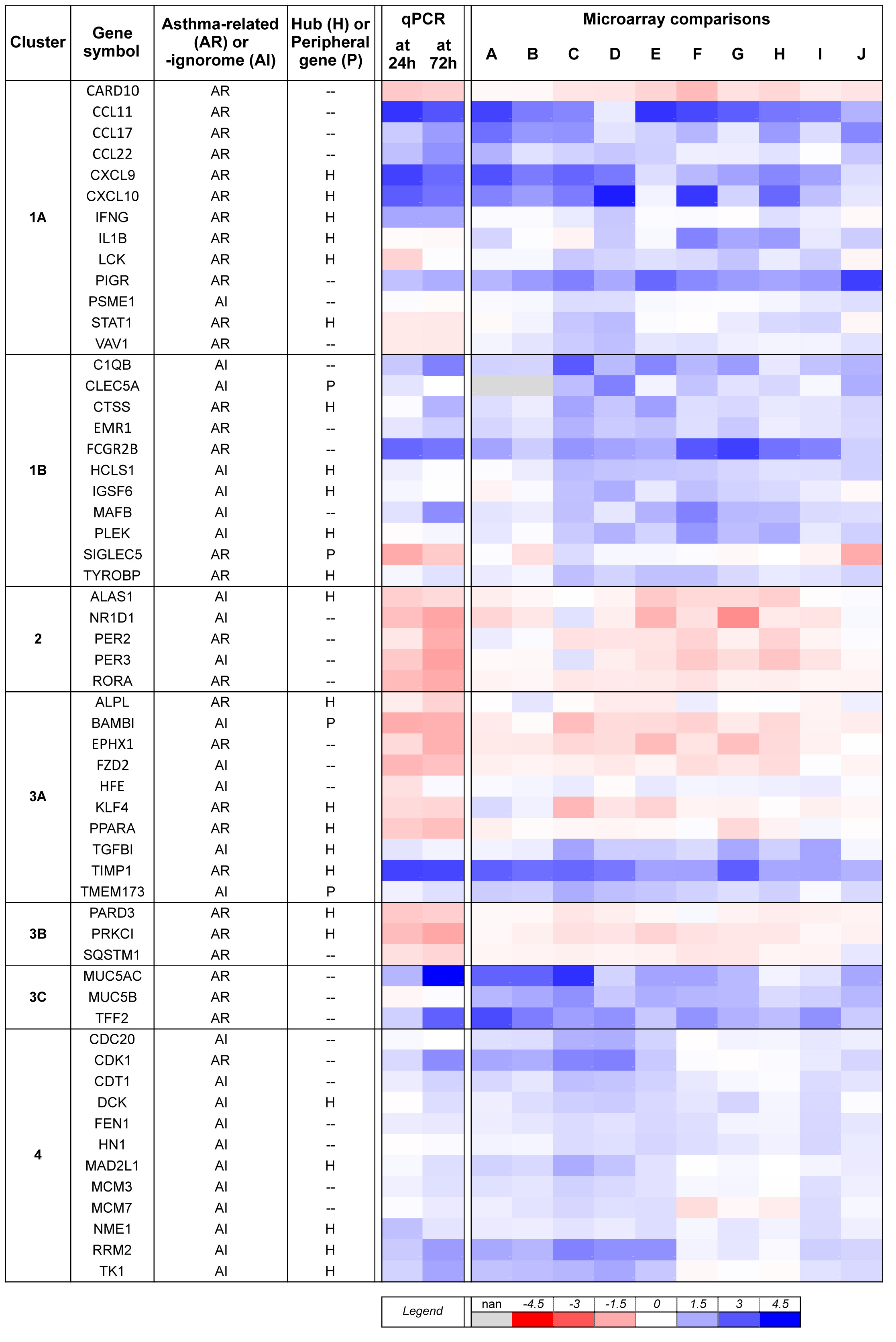


**Supplementary Figure S3.** Expression profiles of 59 selected genes from 7 clusters were confirmed with qPCR. Data are presented as the mean log_2_ fold changes of gene expression by RT- qPCR and microarray relative to control mice. RNA were measured in whole lung extracts and data are representative from 2 independent experiments (*n*=3). Microarray data are from 6 publicly available datasets broken down into 10 direct comparisons of asthmatic and control mice (Table 1 and Fig. 1).

**Supplementary Figure S4**. BioGPS expression heatmaps of genes in 7 main clusters indicates enrichment for genes involved in, 1) leukocytes compared to lung tissues, which indicates that genes in cluster 1A and 1B mainly have a role in inflammation, 2) genes overexpressed in the lung tissues compared to leukocytes, which suggests involvement in the lung tissue response to allergic inflammation (cluster 3A); and genes highly overexpressed in B lymphoblasts, CD105^+^ Endothelial cells, CD34^+^ cells and CD71^+^ early erythroid cells (cluster 4). No specific tissue was found enriched in clusters 2, 3B and 3C, probably due to low number of genes in each cluster. Data are shown as expression levels normalized in log scale within selected set of 17 different tissues (12 leukocytes and 5 present in whole lungs). Euclidean distance was used for clustering genes.

**
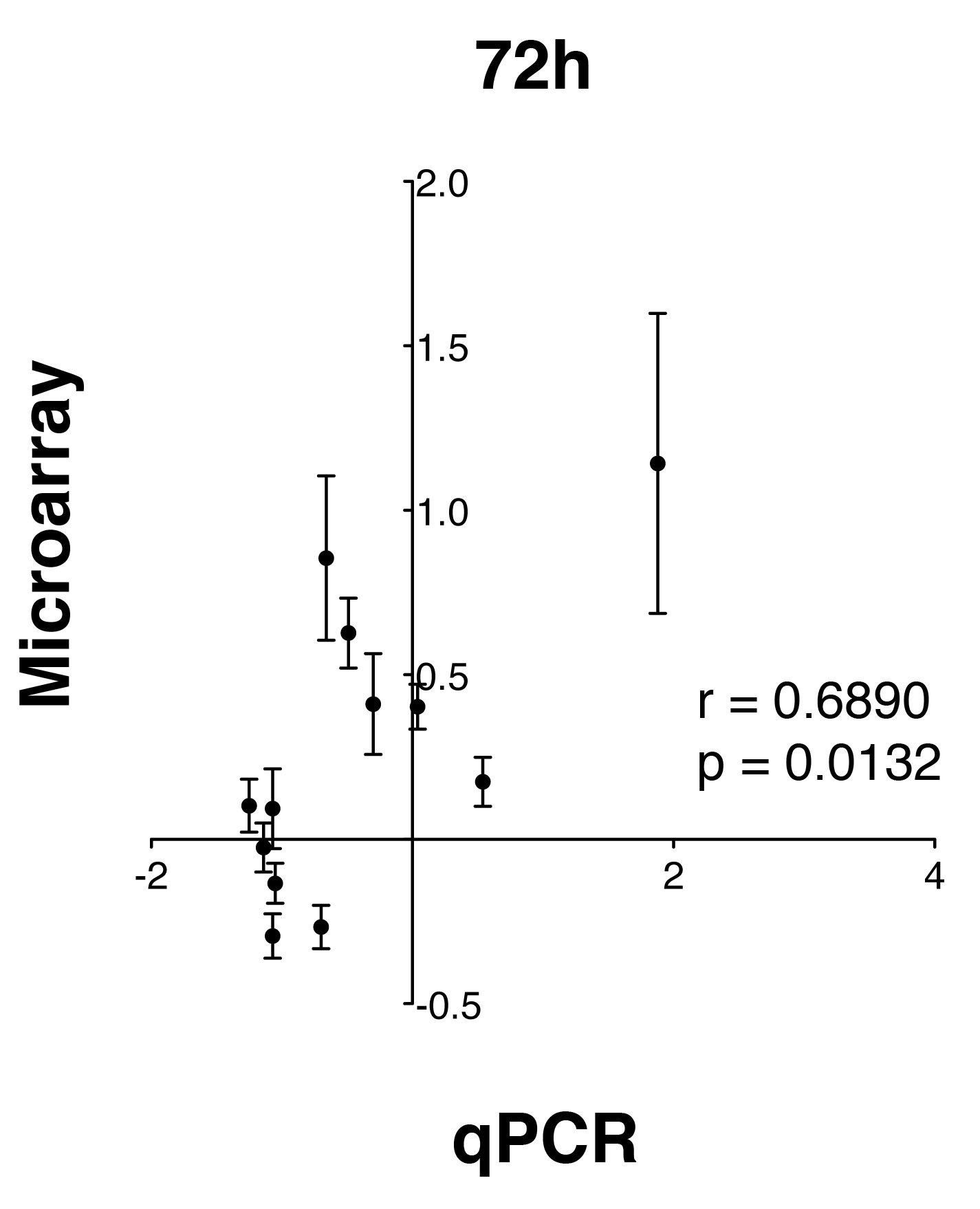
**

**Supplementary Figure S5.** Microarray data for 12 super-connector genes from 10 comparisons of control vs. asthmatic mice correlate to qPCR data from an independent mouse asthma model. For qPCR, OVA-sensitized BALB/c mice received PBS (controls) or OVA challenge 72h before extraction of total lung RNA. Pearson r coefficients and *p* values for each correlation are indicated.


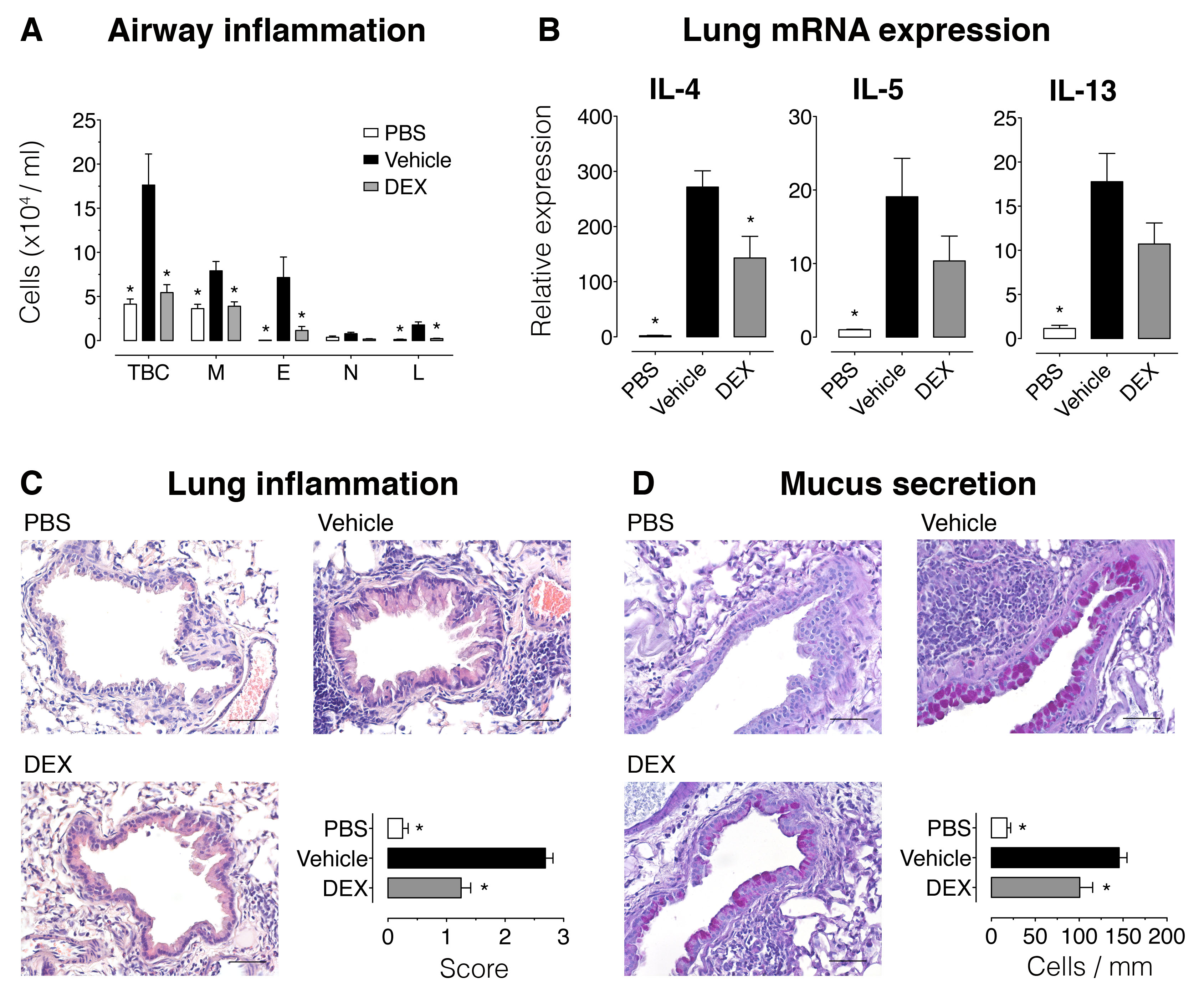


**Supplementary Figure S6.** Dexamethasone significantly inhibits airway and lung inflammation, mucus hypersecretion and lung cytokine mRNA expression. (a) Total and differential cell counts in bronchoalveolar lavage fluid of control (PBS challenged), vehicle and DEX-treated mice 72h after the last aerosol challenge. T – total cells, M – macrophages, E – eosinophils, N – neutrophils, L – lymphocytes. (b) DEX treatment suppressed IL-4, IL-5, and IL-13 mRNA expression in whole lungs at 72h after OVA challenge. Data are expressed as relative difference to corresponding PBS challenged mice. (c) Representative photomicrographs of H&E stained lung sections (scale bars: 50 µm, original objective: 40x). Control animals (PBS) have normal lung physiology, while dense eosinophilic inflammatory infiltrates in vehicle-treated mice and are reduced in DEX-treated group. Grades for lung inflammation were done according to the description in material and methods. (d) Representative photomicrographs of Periodic-acid Schiff (PAS) stained lung sections with fuchsia-stained mucus in epithelial goblet cells (scale bars: 50 µm, original objective: 40x) and numbers of mucus-positive cells per millimeter of basement membrane. Data are shown as mean ± SEM and are pooled from 2 independent experiments (*n* = 6). * *p* < 0.05 *vs.* vehicle treated group, one-way ANOVA followed by Tukey’s multiple comparison test.


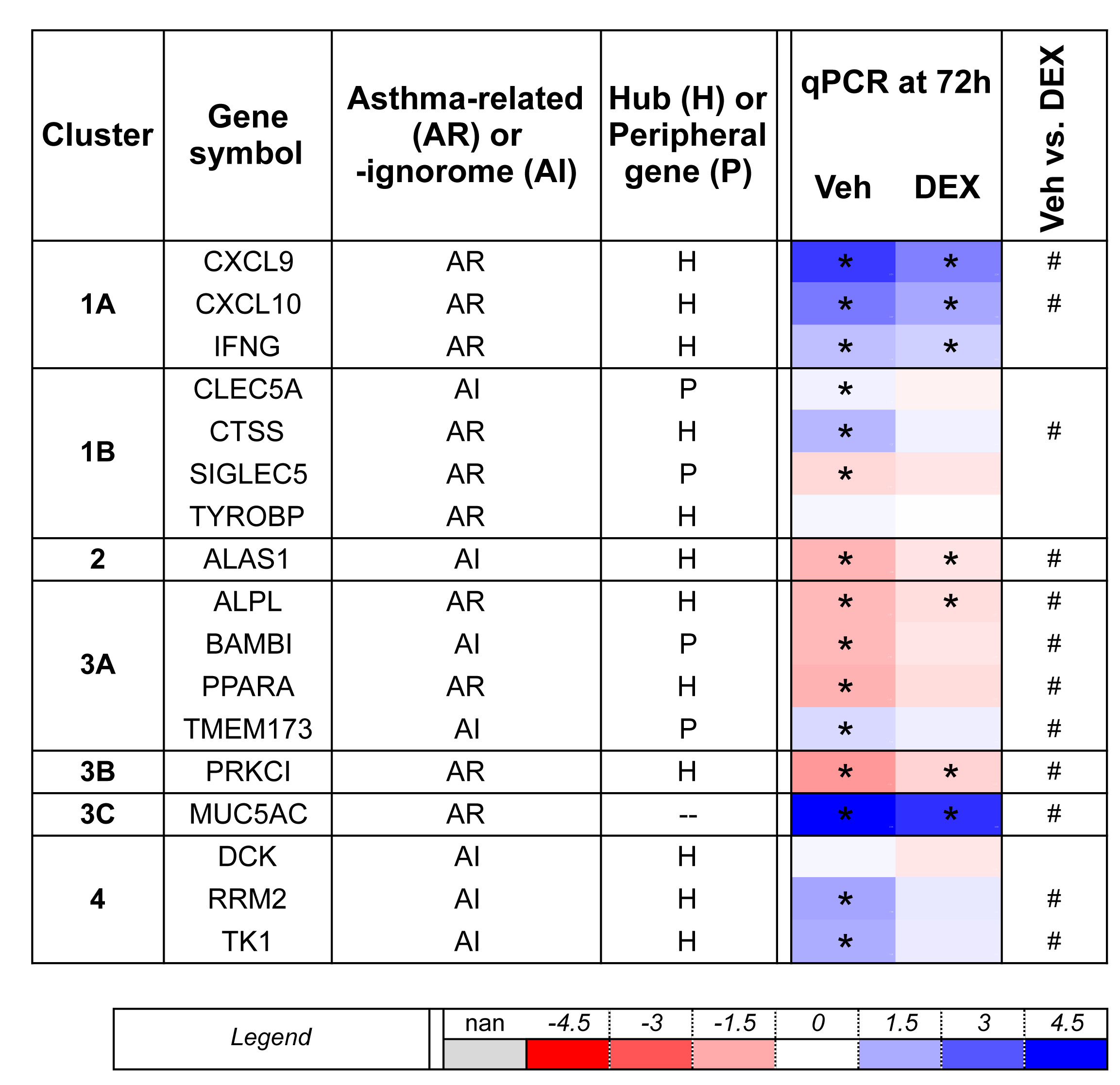


**Supplementary Figure S7.** Expression of randomly selected 12 hubs and 4 peripheral genes in acute allergic asthma after DEX treatment. Total lung RNA was extracted from mice intranasally challenged with PBS (controls) and mice that received vehicle or DEX before and after OVA challenge to induce allergic asthma. Samples were collected at 72h after allergen challenge and gene expression was determined with quantitative PCR. Data are presented as mean log_2_ fold changes of gene expression relative to control (PBS) mice and are pooled from 2 independent experiments (n=6). * *p* < 0.05 compared with PBS challenged mice; # *p* < 0.05 compared with vehicle-treated group (unpaired *t*-test).

**Supplementary Figure S8.** Pathway-driven approach: 76 enriched Gene Ontology Biological Processes (GO.BP) terms in 10 microarray comparisons of asthmatic mice *vs.* controls. GO.BP terms are clustered for semantic similarity: red-yellow zones refer to related biological processes.

**Supplementary table S1.**

Provided as a separate excel document.

**Supplementary table S2.** List of hub and peripheral genes related to acute asthma ignorome.

| **Gene Name** | **Network role** | **Functional Domain** | **Published in “inflammation & immunity”** |
| --- | --- | --- | --- |
| *C1QA* | Hub | Inflammation | Yes |
| *IFITM* | Hub | Inflammation | Yes |
| *HCLS1* | Hub | Inflammation | Yes |
| *IGSF6* | Hub | Inflammation | No |
| *PLEK* | Hub | Inflammation | No |
| *SLC15A3* | Hub | Inflammation | No |
| *CLEC5A* | Peripheral | Inflammation | Yes |
| *COTL1* | Peripheral | Inflammation | Yes |
| *MTPN* | Peripheral | Inflammation | No |
| *PRICKLE1* | Peripheral | Inflammation | No |
| *SDPR* | Peripheral | Inflammation | No |
| *GSTA3* | Hub | Lung-specific insult response | No |
| *CYP2B6* | Hub | Lung-specific insult response | Yes |
| *TGFBI* | Hub | Lung-specific insult response | Yes |
| *COL1A2* | Hub | Lung-specific insult response | Yes |
| *FGF1* | Hub | Lung-specific insult response | Yes |
| *BEX1* | Peripheral | Lung-specific insult response | No |
| *EFHD2* | Peripheral | Lung-specific insult response | No |
| *BAMBI* | Peripheral | Lung-specific insult response | Yes |
| *CHST2* | Peripheral | Lung-specific insult response | Yes |
| *ITGB1BP2* | Peripheral | Lung-specific insult response | Yes |
| *MYO1B* | Peripheral | Lung-specific insult response | Yes |
| *TFPI2* | Peripheral | Lung-specific insult response | Yes |
| *TMEM173* | Peripheral | Lung-specific insult response | Yes |
| *TSPAN7* | Peripheral | Lung-specific insult response | Yes |

**Supplementary Table S3:** Characterization of topological clusters returned by STRINGdb query to STRING database. P-values in the last column are calculated with Wilcoxon rank-sum test on the “internal” and “external” degrees of a cluster.

|  | *Proteins* | *Interactions* | *Expected interactions* | *p-value* |
| --- | --- | --- | --- | --- |
| *Cluster1* | 82 | 1356 | 170 | 0 |
| *Cluster 2* | 114 | 792 | 33 | 0 |
| *Cluster 3* | 282 | 1192 | 289 | 0 |
| *Cluster 4* | 239 | 2723 | 440 | 0 |
| *Cluster 5* | 19 | 24 | 0 | 0 |
| *Cluster 6* | 8 | 8 | 0 | 4.22x10^-14^ |
| *Cluster 7* | 4 | 3 | 0 | 3.17x10^-7^ |
| *Cluster 8* | 15 | 36 | 0 | 0 |

**Supplementary table S4.** List of primers (5’ -> 3’) used for chemokine expression analysis. Primer sequences were mainly selected from PrimerBank (<http://pga.mgh.harvard.edu/primerbank/>).

| **Gene** | **NCBI Gene ID** | **Forward primer** | **Reverse primer** |
| --- | --- | --- | --- |
| HPRT | 15452 | TTGCTCGAGATGTGATGAAGGA | AAAGTTGAGAGATCATCTCCACCAA |
| β-Actin | 11461 | GTGACGTTGACATCCGTAAAGA | GCCGGACTCATCGTACTCC |
| GAPDH | 14433 | ACCCAGAAGACTGTGGATGG | CACATTGGGGGTAGGAACAC |
| ALAS1 | 11655 | TCGCCGATGCCCATTCTTATC | GGCCCCAACTTCCATCATCT |
| ALPL | 11647 | GTGACTACCACTCGGGTGAAC | CTCTGGTGGCATCTCGTTATC |
| BAMBI | 68010 | CATTGCTGGCGGACTGATCTT | CTTGCCCCTTCTTGGAATGGT |
| CADM1 | 54725 | GAACCAGCAGTTCACGATTCT | AGCAAGCATAGCATGGCAAAC |
| CD44 | 12505 | TCTGCCATCTAGCACTAAGAGC | GTCTGGGTATTGAAAGGTGTAGC |
| CLEC5A | 23845 | TTATTGGTTTGGTACGTCAGCC | TGGTCCTGATTGGTAACATTGC |
| CTSS | 13040 | TAGAGGCAGACGCTTCCTATC | CGGGAGCTGAATGTACCTTGA |
| CXCL10 | 15945 | CCAAGTGCTGCCGTCATTTTC | GGCTCGCAGGGATGATTTCAA |
| CXCL9 | 17329 | GGAGTTCGAGGAACCCTAGTG | GGGATTTGTAGTGGATCGTGC |
| DCK | 13178 | TGGCAAGCTCAAAGATGCAGA | AGAAGCGAAAATGTACCTGTCAC |
| ENPP1 | 18605 | GAGTGTCCAGCAGAGTTTGAAT | CACCCCAGGTGTGCAAATACT |
| ERBB2 | 13866 | GAGACAGAGCTAAGGAAGCTGA | ACGGGGATTTTCACGTTCTCC |
| HCLS1 | 15163 | GGCCACGAGTATGTTGCTGAT | CCCTCTCAACTCCATATTTGCC |
| IFNγ | 15978 | ATGAACGCTACACACTGCATC | CCATCCTTTTGCCAGTTCCTC |
| IGSF6 | 80719 | TAGAAGTGGACTACGGTTCTGAC | GTCTGCCTCATTTCTGCATCC |
| IL13 | 16163 | GGATATTGCATGGCCTCTGTAAC | AACAGTTGCTTTGTGTAGCTGA |
| IL1β | 16176 | TTCAGGCAGGCAGTATCACTC | GAAGGTCCACGGGAAAGACAC |
| IL4 | 16189 | ACTTGAGAGAGATCATCGGCA | AGCTCCATGAGAACACTAGAGTT |
| IL5 | 16191 | CTCTGTTGACAAGCAATGAGACG | TCTTCAGTATGTCTAGCCCCTG |
| IL6 | 16193 | TAGTCCTTCCTACCCCAATTTCC | TTGGTCCTTAGCCACTCCTTC |
| KLF4 | 16600 | ATCCTTTCCAACTCGCTAACCC | CGGATCGGATAGCTGAAGCTG |
| LCK | 16818 | AACTTCGTGGCGAAAGCAAAC | CTGACCGACAGGGAAAAGGAC |
| MAD2L1 | 56150 | GTGGGAAGAATCGGGACCG | CAGTCATTGACAGGGGTTTTGT |
| MUC5AC | 17833 | GTGGTTTGACACTGACTTCCC | CTCCTCTCGGTGACAGAGTCT |
| MUC5B | 74180 | AAGCTGCCCTACAGTCGTG | GACTAGGCGGATGCTGATTTT |
| NME1 | 18102 | AGGAGCACTACACTGACCTGA | GGTTGGTCTCTCCAAGCATCA |
| NR1D1 | 217166 | GCTCAGCGTCATAATGAAGCG | GGGCCGAATATACGTGGGT |
| NR3C1 | 14815 | CCGGGTCCCCAGGTAAAGA | TGTCCGGTAAAATAAGAGGCTTG |
| PARD3 | 93742 | ACATGCTGATACCGGATTGGA | CACTGAAAGGCACTACATGGATT |
| PER2 | 18627 | CAGGTTGAGGGCATTACCTCC | AGGCGTCCTTCTTACAGTGAA |
| PER3 | 18628 | TCAAGACGTGAGGGCGTTCTA | CATTCATACTGCGAGGCTCTTT |
| PLEK | 56193 | TTCAGAGGGGTTATCATCAAGCA | CCCAGCAGGATCATAGTAGTGC |
| PPARA | 19013 | TACTGCCGTTTTCACAAGTGC | AGGTCGTGTTCACAGGTAAGA |
| PRKCD | 18753 | TGGGGGTGACCTGATGTTC | CCAGCACCAACAATACCTGTAA |
| PRKCI | 18759 | CCACACTTTTCAAGCCAAACG | TGCACTTGTATCCTTGTCGTC |
| RELA | 19697 | AGCGCGGGGACTATGACTT | GCCCGGTTATCAAAAATCGGAT |
| RORA | 19883 | GATGACCTCAGCACCTATATGGA | CGGGTTTGATCCCATTGATGTC |
| RRM2 | 20135 | GGAAGCTCTGAAACCCGATGA | ACTTCTTGGCTAAATCGCTCC |
| SIGLEC5 | 233186 | TGCACAGAAAATCGACACAGG | TCAGAGCTATCACAAGCACAGA |
| STAT1 | 20846 | GCTGCCTATGATGTCTCGTTT | TGCTTTTCCGTATGTTGTGCT |
| TFF2 | 21785 | CCTTGGTGTTTCCACCCACTT | AGCAGCAGTTTCGACTGGC |
| TGFBI | 21810 | TGTCCTGGATATGAAAAGGTCCC | GGTGGTCGATCCCACAACTC |
| TIMP1 | 21857 | GCAACTCGGACCTGGTCATAA | CGGCCCGTGATGAGAAACT |
| TK1 | 21877 | AAGCTGCCTACACGAAGAGG | CGGCACACGGAGTGATACTT |
| TMEM173 | 72512 | CTACATTGGGTACTTGCGGTT | GCACCACTGAGCATGTTGTTATG |
| TRAF6 | 22034 | AAAGCGAGAGATTCTTTCCCTG | ACTGGGGACAATTCACTAGAGC |
| TYROBP | 22177 | GAGTGACACTTTCCCAAGATGC | CCTTGACCTCGGGAGACCA |
| VWF | 22371 | CTTCTGTACGCCTCAGCTATG | GCCGTTGTAATTCCCACACAAG |

**Supplementary references**

1. Edgar, R., Domrachev, M. & Lash, A. E. Gene Expression Omnibus: NCBI gene expression and hybridization array data repository. *Nucleic Acids Res.* **30,** 207–210 (2002).

2. Gentleman, R. C. *et al.* Bioconductor: open software development for computational biology and bioinformatics. *Genome Biol.* **5,** R80 (2004).

3. Irizarry, R. A. *et al.* Exploration, normalization, and summaries of high density oligonucleotide array probe level data. *Biostatistics* **4,** 249–264 (2003).

4. Benjamini, Y. & Hochberg, Y. Controlling the false discovery rate: a practical and powerful approach to multiple testing. *Journal of the Royal Statistical Society. Series B Methodological* **57,** 289–300 (1995).

5. Franceschini, A. *et al.* STRING v9.1: protein-protein interaction networks, with increased coverage and integration. *Nucleic Acids Res.* **41,** D808–15 (2013).

6. Saito, R. *et al.* A travel guide to Cytoscape plugins. *Nat. Methods* **9,** 1069–1076 (2012).

7. Dennis, G. *et al.* DAVID: Database for Annotation, Visualization, and Integrated Discovery. *Genome Biol.* **4,** P3 (2003).

8. Yu, G. *et al.* GOSemSim: an R package for measuring semantic similarity among GO terms and gene products. *Bioinformatics* **26,** 976–978 (2010).

9. Riba, M. *et al.* A strong anti-inflammatory signature revealed by liver transcription profiling of Tmprss6-/- mice. *PLoS ONE* **8,** e69694 (2013).

10. Hong, F. *et al.* RankProd: a bioconductor package for detecting differentially expressed genes in meta-analysis. *Bioinformatics* **22,** 2825–2827 (2006).

11. Hong, F. & Breitling, R. A comparison of meta-analysis methods for detecting differentially expressed genes in microarray experiments. *Bioinformatics* **24,** 374–382 (2008).

12. Maglott, D., Ostell, J., Pruitt, K. D. & Tatusova, T. Entrez Gene: gene-centered information at NCBI. *Nucleic Acids Res.* **39,** D52–7 (2011).

13. Chen, J., Bardes, E. E., Aronow, B. J. & Jegga, A. G. ToppGene Suite for gene list enrichment analysis and candidate gene prioritization. *Nucleic Acids Res.* **37,** W305–11 (2009).

14. Rappaport, N. *et al.* MalaCards: A Comprehensive Automatically-Mined Database of Human Diseases. *Curr Protoc Bioinformatics* **47,** 1.24.1–1.24.19 (2014).

15. Stelzer, G. *et al.* In-silico human genomics with GeneCards. *Hum. Genomics* **5,** 709–717 (2011).

16. Csardi, G. & Nepusz, T. The igraph software package for complex network research. *InterJournal, Complex Systems* 1695 (2015).

17. Bertolazzi, P., Bock, M. E. & Guerra, C. On the functional and structural characterization of hubs in protein-protein interaction networks. *Biotechnol. Adv.* **31,** 274–286 (2013).

18. Chen, E. Y. *et al.* Enrichr: interactive and collaborative HTML5 gene list enrichment analysis tool. *BMC Bioinformatics* **14,** 128 (2013).

19. Wu, C. *et al.* BioGPS: an extensible and customizable portal for querying and organizing gene annotation resources. *Genome Biol.* **10,** R130 (2009).

20. Bosnjak, B. *et al.* Tiotropium bromide inhibits relapsing allergic asthma in BALB/c mice. *Pulmonary Pharmacology & Therapeutics* **27,** 44–51 (2014).
